# Acquired tracheoesophageal fistula due to high intracuff pressure

**DOI:** 10.4103/1817-1737.37950

**Published:** 2008

**Authors:** Akmal A. Hameed, Hasan Mohamed, Motasem Al-Mansoori

**Affiliations:** Department of ICU, Salmaniya Medical Complex, Kingdom of Bahrain; * *Department of Anesthesia, Salmaniya Medical Complex, Kingdom of Bahrain*

**Keywords:** Cuff leak, endotracheal tube, intracuff pressure, tracheal damage, tracheoesophageal fistula

## Abstract

High-compliance endotracheal tube cuffs are used to prevent gas leak and also pulmonary aspiration in mechanically ventilated patients. However, the use of the usual cuff inflation volumes may cause tracheal damage and lead to tracheoesophageal fistula.

Tracheostomy tube cuffs seal against the tracheal wall and prevent leakage of air around the tube, assuring that the tidal volume is delivered to the lungs. In the past, high-pressure cuffs were used, but these contributed to tracheal injury and have been replaced by high-volume, low-pressure cuffs. For long-term applications, some newer tubes have low-profile (tight to shaft) cuffs that facilitate the tracheostomy tube changes by eliminating the lip that forms when standard cuffs are deflated.

## Introduction

Tracheostomy tube cuff volumes and pressures require constant monitoring to avoid tracheal injury. Past literature recommended routine inflation and deflation of cuffs every few hours; but this has not been shown to reduce the risk of tracheal injury, and it actually increases the risk for aspiration.[1] Because cuff pressures >30 cmH_2_O compress mucosal capillaries and impair blood flow, with total occlusion occurring at 50 cmH_2_O, it is generally recommended that cuff pressures do not exceed 20 cmH_2_O. However, monitoring cuff pressure alone is insufficient, because tracheal damage and increases in cuff volume can occur even when cuff pressures are maintained within the desired range. Cuff volumes should not exceed 6 to 8 ml, ideally; and the need to inflate the cuff to > 10 ml should raise concerns about tracheal injury.[2]

High-compliance endotracheal tube cuffs are used to prevent gas leak and also pulmonary aspiration in mechanically ventilated patients. However, the use of the usual cuff inflation volumes produces transmission of the pressure directly to the tracheal wall around the cuffs. When the cuff pressure is over 40 cmH_2_O, which is the perfusion pressure of the tracheal mucosa and submucosa,[3] loss of mucosal ciliar,[4–5] ulceration, bleeding,[5] tracheal stenosis[6] and tracheoesophageal fistula[7] may occur.

## Case Report

A 35-year-old male was admitted to intensive care unit through accident/emergency after a road traffic accident and having sustained multiple injuries. He was a drug addict, alcoholic, hepatitis B and C positive. He was intubated for airway protection and transferred to ICU. He had fracture 3 and 4 cervical vertebra complete transaction of spinal cord at C4 level (quadriplegic); fracture 4, 5, 6 ribs on the right side; and bilateral pneumothorax (bilateral chest tubes inserted); rupture spleen and liver.

He was taken to the operating room and spleenectomy and hepatic repair were done; and keeping in mind the seriousness of injuries and anticipating long recovery time, the elective surgical tracheostomy was done in operating room.

**Figure 1:**
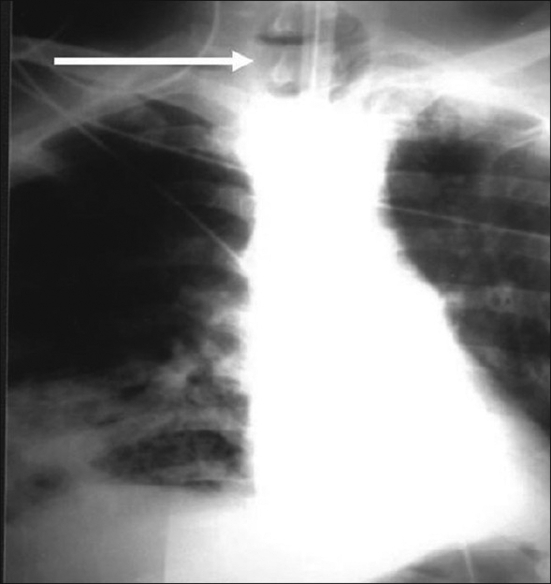
The arrow in chest radiograph shows tracheoesophageal fistula

The patient was shifted back to the ICU on ventilator. He was started on antibiotics, and supportive care was given and the feeding was started by nasogastric tube. Multiple attempts of extubation failed; and on day 15, the assigned staff nurse noticed bubbling and air leak from the mouth and the patient started desaturating. He was febrile, with white blood cell level increasing. On suctioning through endotracheal tube, food material was noticed. Chest radiograph showed opacities in the right lower lung region and large endotracheal cuff shadow [Figure 1]. Bronchoscopy revealed the fistula and demonstrated the passage of secretions and air. Intracuff pressure was measured and was found to be 40 cmH_2_O. Water dye was given through mouth, which was traced into the trachea. He was diagnosed as having tracheoesophageal fistula.

Appropriate antibiotic was started for aspiration pneumonia, and the patient improved with regard to his infection but later expired.

## Discussion

Tracheoesophageal fistulae are severe lesions that lead to serious and often fatal pulmonary complications. These lesions are predominantly iatrogenic, occurring in the course of tracheal intubation for resuscitation; or are of malignant origin with invasion of esophageal and tracheal walls.[8]

Tracheobronchial injuries may be caused by external injuries from blunt or penetrating trauma or by internal injuries subsequent to inhalation of fumes or gases or aspiration of liquids or foreign bodies.[9] These injuries are rare but can be life threatening. Acquired, nonmalignant tracheoesophageal fistulae usually result from erosion of the tracheal and esophageal walls by endotracheal or tracheostomy tube cuffs, especially when a rigid nasogastric tube is in place. This life-threatening condition is infrequent with the use of high-volume and low-pressure cuffs.

Higher intracuff pressure is needed to maintain the cuff's sealing effect in intubated patients. Prolonged intubation and the presence of a wide-bore gastric tube cause pressure on the sandwiched mucosa between the cuff of the tracheal tube and the gastric tube. Excessive motion of the tracheal tube during frequent dressing changes and respiratory care is another predisposing factor. Local infection worsens the mucosal damage, resulting in perforation. The duration of artificial ventilation by means of a tracheal tube in these patients exerts a basic influence on the occurrence of this complication.

In our reported case, tracheoesophageal fistula occurred due to high intracuff pressure; because when high intracuff pressure was measured after noticing the TEF, it was 40 cmH_2_O.

There have been descriptions of the occurrence of tracheal sequelae, secondary to endotracheal tube cuff high pressure, since mechanical ventilation was introduced in 1950. The first endotracheal tube cuffs used were made of low-compliance latex rubber, requiring high pressure levels to seal the tracheal lumen. Since the end of the 1970s, these endotracheal tube cuffs have been replaced by ones made of high-compliance plastic. Thus, higher air volumes can be injected with only small pressure increases, decreasing the incidence of tracheal lesions.[7] Low-pressure endotracheal tube cuffs present wider support surfaces on the tracheal mucosa than do the high-pressure ones; and therefore, the pressure exerted by the former is lower.

Ischemic damage of the trachea depends on the balance between mucosal perfusion pressure and the pressure exerted by the cuff. When the cuff pressure exceeds tracheal mucosal perfusion pressure, induction of ischemia and / or necrosis will just be a question of time.[3] Tracheitis without ulceration is the initial lesion that occurs, followed by mucosal denudation and exposure of trachea cartilage.[3] Other associated factors can increase the incidence of post-intubation tracheal complications, even with cuff pressures that appear not be excessive on the tracheal mucosa. Among these factors are included the decrease in mucosal blood flow produced by hypotension, shock and anemia[4] and low oxygen delivery to tracheal tissue produced by hypoxemia, anemia and metabolic acidosis. Additional mechanical factors, such as the placement of nasogastric tubes, seem to increase the risk of developing tracheoesophageal fistulae in patients under longtime tracheal intubations.[6]

Tracheal stenosis occurs in response to tube-related trauma at various levels of the trachea, including the suprastomal area, the stoma itself, the tube cuff and the distal tip of the tube tip. The cuff site was, at one time, a common location for stenosis; but after the advent of low-pressure, high-volume cuffs for both endotracheal and tracheostomy tubes, the stoma site is now more frequently affected.

In the critical care setting, tracheal stenosis occurs in 0–16% of patients who have tracheostomies, with higher rates occurring after surgical tracheostomies are performed, as compared to percutaneous dilatational tracheostomy. The incidence in patients who require long-term ventilation is unknown because of a lack of studies performed. Patients are usually asymptomatic until the tracheal diameter is reduced to less than 5 mm, at which time they may present with dyspnea, cough, stridor and inability to clear secretions. These symptoms may not be apparent until trials with speaking valves and full capping are undertaken.

Patients who present with stridor should be evaluated for vocal cord dysfunction, tracheal stenosis and malacia and a tube that excessively blocks the trachea despite cuff deflation. Measures to prevent tracheal stenosis include the inflation of cuffs, when necessary; maintenance of intracuff pressures <20 cmH_2_O, using properly sized tracheostomy tubes; and avoidance of excessive pressure of the tube tip on either the anterior or posterior tracheal wall.

## Conclusion

Endotracheal tube cuff pressures in intensive care units are routinely high and are significantly higher. Endotracheal tube cuff pressures should be routinely measured by manometer to minimize trauma to the tracheal mucosa and surrounding structures.

Many different tracheostomy tubes are available that are constructed of different materials; in different sizes, shapes and cuff design. Tubes should be selected with care, based on the patient's body habitus, ventilatory capacity and aspiration risk. Regardless of the tube chosen, every effort should be made to preserve normal speech and swallowing, as long as the patient is capable of these functions. Management of patients who have tracheostomies aims to minimize complications by avoiding overinflation of the tube cuff, even by using cuffless tubes if possible and eschewing deep suctioning. In appropriately selected patients receiving optimal management, tracheostomy ventilation can achieve long-term survival (up to several decades in patients with slowly progressive neuromuscular conditions) with an acceptable quality of life.
